# Mechanisms Underlying Metabolic Syndrome-Related Sarcopenia and Possible Therapeutic Measures

**DOI:** 10.3390/ijms20030647

**Published:** 2019-02-02

**Authors:** María Esther Rubio-Ruiz, Verónica Guarner-Lans, Israel Pérez-Torres, María Elena Soto

**Affiliations:** 1 Department of Physiology, Instituto Nacional de Cardiología “Ignacio Chávez”, Juan Badiano 1, Sección XVI, Tlalpan, Mexico City 14080, Mexico; esther.rubio@cardiologia.org.mx; 2 Department of Pathology, Instituto Nacional de Cardiología “Ignacio Chávez”, Juan Badiano 1, Sección XVI, Tlalpan, Mexico City 14080, Mexico; israel.perez@cardiologia.org.mx; 3 Department of Immunology, Instituto Nacional de Cardiología “Ignacio Chávez”, Juan Badiano 1, Sección XVI, Tlalpan, Mexico City 14080, Mexico; elena.soto@cardiologia.org.mx

**Keywords:** sarcopenia, metabolic syndrome, skeletal muscle, obesity, sarcopenia treatment

## Abstract

Although there are several reviews that report the interrelationship between sarcopenia and obesity and insulin resistance, the relation between sarcopenia and the other signs that compose the metabolic syndrome (MetS) has not been extensively revised. Here, we review the mechanisms underlying MetS-related sarcopenia and discuss the possible therapeutic measures proposed. A vicious cycle between the loss of muscle and the accumulation of intramuscular fat might be associated with MetS via a complex interplay of factors including nutritional intake, physical activity, body fat, oxidative stress, proinflammatory cytokines, insulin resistance, hormonal changes, and mitochondrial dysfunction. The enormous differences in lipid storage capacities between the two genders and elevated amounts of endogenous fat having lipotoxic effects that lead to the loss of muscle mass are discussed. The important repercussions of MetS-related sarcopenia on other illnesses that lead to increased disability, morbidity, and mortality are also addressed. Additional research is needed to better understand the pathophysiology of MetS-related sarcopenia and its consequences. Although there is currently no consensus on the treatment, lifestyle changes including diet and power exercise seem to be the best options.

## 1. Introduction

Skeletal muscle is the most abundant tissue constituting around half of the human body mass and many physiological functions depend on it. It generates power, which determines human mobility, and participates in maintaining homeostasis-exerting metabolic functions. Any deterioration in the contractile tissue and metabolic properties of skeletal muscle has an important effect on human health [1]. Nevertheless, muscle mass and functions are not routinely assessed [2].

Age-related losses in skeletal muscle mass and function have been widely studied and are an important current and future public health issue since the average life span has increased in recent years. However, its association with cardiometabolic diseases has been less studied. Sarcopenia comprises both muscle loss and its dysfunction, which induce contractile impairment and metabolic and endocrine abnormalities. It affects whole-body metabolism and the immune/inflammatory response, and includes parameters related to force, functional capacity, and the amount of body fat [2,3]. Sarcopenia is a multifactorial process in whose development the genetic predisposition plays an important role. It can be attributed to a combination of “neural” and “muscular” factors and is related to physical activity, nutritional intake, oxidative stress, and hormonal changes [4,5]. Regarding nutritional intake, appetite regulation and physical activity affect energy balance, and decreases in body fat mass and exercise favor muscle health. Inflammation regulates appetite, which influences weight; it induces anorexia and fat loss in combination with sarcopenia in some individuals. However, in others, appetite is maintained, despite activation of systemic inflammation, leading to sarcopenia with normal or increased fat content [2]. After reaching adulthood, humans and most mammals undergo a progressive reduction in the size and number of muscle fibers and in muscular force generated as aging proceeds. This loss process is accompanied with a replacement of muscle by fat and connective tissue [6,7]. The decline in muscle mass begins at about 30 years of age with a significant loss after 60 years, losing approximately 3% (3.6% in men and 2.8% in women) of the muscle strength every year [6,7,8,9].

Sarcopenia is also accompanied by infiltration of muscle tissue by lipids, leading to increased frequency of adipocyte or lipid deposition within muscle fibers. Lipid deposition in muscle (myostatosis) is also the result of the reduced oxidative capacity of muscle fibers with aging [6,10]. Elevated fat mass in combination with low muscle mass, or elevated fat mass together with low muscle strength are termed as sarcopenic obesity. The novel concept of dynapenic obesity takes into account the force of the muscle and anthropometric measurements [11].

Low muscle strength is associated with an increase in metabolic syndrome (MetS), type 2 diabetes, and in some cases, mortality [5]. Here, we review the mechanisms underlying MetS-related sarcopenia and discuss the possible therapeutic measures proposed.

## 2. Metabolic Syndrome and Sarcopenia

Metabolic syndrome (MetS) is a cluster of pathological signs that increases the risk of type 2 diabetes, cardiovascular disease, and cancer. The signs that form part of MetS include: central obesity, insulin resistance, hyperglucemia, dyslipidemia, inflammation, and hypertension, among others. Some of these signs have been associated with sarcopenia, particularly obesity and insulin resistance.

Sarcopenia and MetS have negative repercussions in the quality of life of elderly people, being the cause of weakness, dependence, and an increase in morbi-mortality rates [12]. Furthermore, a reduced muscle mass in aged individuals has been associated with decreased survival rates following critical illness [13]. Nevertheless, individuals that show simultaneously obesity and probably other signs of MetS and sarcopenia are at an increased risk of several health events compared with those who show one of the signs of the syndrome or sarcopenia alone [14].

There is a positive feedback loop between sarcopenia and obesity [15,16]. Due to this synergistic action of adipose and muscular tissues in elderly people, a new and important concept from the health viewpoint has emerged: sarcopenic obesity. Although the first mention of this concept appeared in a study published by Heber et al. [17], it is not until several years later when it is more clearly defined. According to Baumgartner et al. [4], sarcopenic obesity is defined as the simultaneous presence of a muscular mass below two standard deviations of the mean for a young population (<7.26 kg/m^2^ in males and <5.45 kg/m^2^ in females) and a percentage of body fat above the median (>27% in males and >38% in females). However, there is not a consensus in the way of calculating it since it is a novel concept and depends on the equation used; therefore, there are variations in the results obtained in different studies [10,18,19]. Furthermore, the syndrome of sarcopenic obesity is poorly defined clinically; Cleasby [14] and some authors have even changed its definition, naming it obesity sarcopenia to reflect the direction of the pathological pathway [15].

The association with the functional status impairment was stronger for sarcopenic obesity than for either obesity or sarcopenia alone. Baumgarther et al. [4] found that subjects having sarcopenic obesity had a 2-fold increase in the risk of development of discapacity or functional limitation in their instrumental every day activities than subjects having obesity or sarcopenia alone. However, other transversal studies with elderly women showed no relation between sarcopenic obesity and functional limitation, even if there was an association with obesity and functional decline [20].

Although there are several reviews that report the interrelationship between sarcopenia and obesity and insulin resistance, the relation between sarcopenia and the other signs that constitute the MetS has not been extensively revised. Some authors have proposed that insulin resistance might be the underlying cause of the association between obesity and sarcopenia, and fatty acids and triglycerides are in turn related to insulin resistance [14]. Additional research is needed to better understand the pathophysiology of MetS-related sarcopenia and its consequences, and although new proposals for possible treatments have been forwarded, many of them have not been tested.

### 2.1. Obesity, Inflammation, and Insulin Resistance

Previous studies have analyzed the association of the changes in skeletal muscle mass or the ratio of muscle mass in relation to adipose tissue and MetS development [21]. There is a relation between the growth of visceral adipose tissue and muscle atrophy that is suggested by the diminished expression of human contractile proteins in myotubes when they are co-cultured with adipocytes extracted from individuals with obesity [22].

In obesity and MetS, adipocytes in the adipose tissue undergo hypertrophy and hyperplasia, and are therefore enlarged. The tissue is also infiltrated by activated pro-inflammatory macrophages and other immune cells. Cells from this modified adipose tissue are characterized by an elevated production of circulating proinflammatory molecules [23,24], as well as by a dysregulated production of various adipokines, such as leptin and adiponectin, that cause deleterious effects of tissues such as the hypothalamus, liver, pancreas, and skeletal muscle [25]. When the adipokine secretion is modified, it can have as a consequence an elevated food intake and a decrease in the energy used through effects in the hypothalamus, and to diminished insulin sensitivity by the muscle [1]. The production of different substances from adipose tissue, such as tumor necrosis factor-α (TNF-α), monocyte chemoattractant protein–1 (MCP-1), interleukin 6 (IL-6), and C-reactive protein (CRP), which are known to influence insulin resistance and the secretion of anabolic hormones, such as insulin and growth hormone (GH), could induce sarcopenic obesity [1,25,26].

TNF-α may play important roles in inflammatory catabolic states, such as obesity and insulin resistance, because it is responsible for the increase in gluconeogenesis, loss of adipose tissue, and proteolysis. TNF-α promotes protein degradation and may be associated with obesity-related skeletal muscle atrophy and age-related sarcopenia. It induces atrophic factors such as MAFbx/atrogin-1 and MuRF1 in myotubes and causes wasting of muscle by the induction of the ubiquitin–proteasome system [27,28].

Higher levels of IL-6 and CRP are associated with a two- to three-fold greater risk of losing more than 40% of grip strength, and predisposition to sarcopenia. Furthermore, lipocalin-2, an adipose-derived cytokine that plays a critical role in the regulation of lipid metabolism, could be a possible candidate regulating the amount of adipose tissue under chronic inflammation and insulin resistance. However, it remains to be elucidated whether lipocalin-2 levels increase with normal aging and are further elevated in MetS-related sarcopenia [1,29]. Moreover, plasminogen activator inhibitor-1(PAI-1), which is derived from adipocytes and mainly from visceral fat, is liberated to the circulation in parallel with the increase in fat mass. It functions as a crucial adipokine that negatively affects the physiological metabolism and the vascular biology. In mice models, PAI-1 is implicated in insulin resistance, osteoporosis, and excess glucocorticoid-induced sarcopenia. The fibrinolisis abnormalities induced by PAI-1 are associated with the MetS, leading to cardiovascular disease through dysregulated vascular clotting, endothelial dysfuction, and metabolic abnormalities. PAI-1 is related with other pathological states such as the non-alcoholic hepatic steatosis and cancer. Therefore, it has been proposed as a biomarker of inflammatory activity in these diseases or as a study target for possible therapeutic agents [30].

In obesity, lipids are also ectopically accumulated in skeletal muscle and other tissues. Adipocyte fatty acid-binding protein (A-FABP) is a circulating biomarker closely associated to MetS and sarcopenia; it is involved in the uptake of fatty acids and their subsequent transport towards the mitochondrial β-oxidation system. Lipid accumulation in muscle is strongly linked to metabolic and inflammatory pathways [31,32]. Muscular lipid accumulation impairs insulin action through locally released adipokines and fat-free fatty acids. In particular, fatty acids can induce pro-inflammatory macrophage activation and p38 mitogen-activated protein kinase (MAPK)-mediated insulin resistance. Insulin resistance could cause muscle wasting through suppression of phosphoinositide 3-kinase (PI3K)/protein kinase B (Akt) signaling [33,34]. Defects that impair insulin-stimulated glucose disposal into skeletal muscle impact whole body glucose metabolisms and homeostasis and protein synthesis. When there is insulin resistance in muscle, the anabolic response to exercise and amino acids is reduced, and ceramide production, which contributes to insulin resistance, is increased by saturated fatty acids [35]. Triacylglycerols and other lipids are transformed to diacylglyerols by adipose triglyceride lipase (ATGL); then, they are hydrolyzed by the hormone sensitive lipase (HSL). The storage of intracellular diacylgyercerols that diminish insulin signaling is increased when the expression of ATGL and HSL is high in skeletal muscle [36]. Indeed, increases in leptin concentration may lead to leptin resistance and thus to a reduction of fatty acid oxidation in muscles that further contributes to ectopic fat deposition [37].

The intramuscular lipids also induce mitochondrial dysfunction characterized by impaired β-oxidation capacity and increased reactive oxygen species (ROS) formation, providing a lipotoxic environment and an increased secretion of some pro-inflammatory myokines that activate cellular stress signaling, apoptosis, and atrophy in skeletal muscle [38,39]. ROS function as second messengers for TNF-α in skeletal muscle and they can directly or indirectly activate nuclear factor kappa B (NF-κB), which increases IL-6 [40,41]. Mitochondrial dysfunction decreases the expression of genes implicated in oxidative phosphorylation, which are regulated through the 1α co-activator of the receptor activated by peroxisome-gamma proliferator (PPAR-γ) (PGC-1α). PGC-1α is activated under oxidative stress and its expression determines mitochondrial biogenesis, the presence of oxidative myofibers and vascularization in skeletal muscle [42]. The disruption of several positive regulators of muscle hypertrophy, such as Akt, plays an important role in the progression of sarcopenia since it reduces protein synthesis [14].

### 2.2. Cross Talk between Muscle and Adipose Tissue

**Figure 1:**
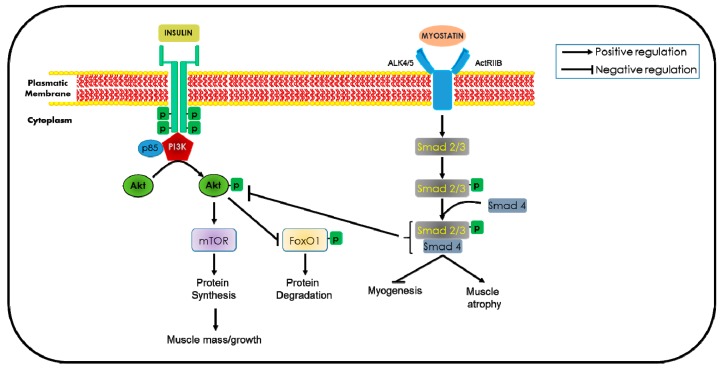
Schematic illustration of myostatin signaling pathway in sarcopenia. Signaling activated by insulin positively regulates muscle mass, downstream of protein kinase Akt and mTOR. Myostatin first binds to the activin receptor (ActRIIB)/Alk 4/5 on skeletal muscle causing phosphorylation of Smad2 and Smad3, and the recruitment of Smad4 into a Smad complex, which leads to muscle atrophy. Second, Smad 2/3/4 complex down-regulate the activity of Akt, thereby inhibiting protein synthesis. Akt blocks FOXO1 nuclear translocation to inhibit protein degradation.

Not only adipose tissue produces and secretes biologically active proteins, but also skeletal muscle produces, expresses, and secretes cytokines and other peptides that exert autocrine, paracrine, or endocrine effects that have been classified as myokines. Myokines contribute to the adaptation to mechanical, neural, and humoral stimuli, and play critical roles in physical activity, energy expenditure, and glucose disposal. Myokines also enhance inflammation in adipose tissue and/or induce chronic low-grade systemic inflammation, establishing a damaging vicious circle that keeps up adipose tissue and skeletal muscle inflammation, thus triggering and supporting the development of sarcopenia [19]. For example, increased expression of myostatin (MSTN), a secreted anabolic inhibitor of muscle growth and development, has been associated with obesity and insulin resistance. MSTN negatively regulates the activity of the Akt pathway, which promotes protein synthesis, and increases the activity of the ubiquitin–proteasome system to induce atrophy [43] (see Figure 1).

Muscle contraction induces the production of many proteins by skeletal tissue modifying the protein secretion pattern that is established during physical inactivity. For example, IL-6 was named a myokine because its levels increased in response to exercise. IL-6 is an important player in metabolism due to its effects on the liver, adipose tissue, and the immune system. Moreover, lipolysis may be stimulated by IL-6, and insulin resistance in muscle may be induced by adipocyte-derived IL-6 [44]. IL-15, in turn, is a myokine expressed by skeletal muscle that has anabolic effects upon muscle. It also participates in the reduction of adipose tissue mass [45,46].

Brain-derived neurotrophic factor (BDNF) is a protein produced in skeletal muscle cells that is increased by contraction, and it regulates neuronal development and controls body mass and fat oxidation via AMP-activated protein kinase (AMPK) [45].

## 3. Sarcopenia and Sexual Dimorphism

Although the exact mechanisms that lead to the loss of muscular mass are still unknown, some of the factors participating in sarcopenia, sarcopenic obesity, and MetS-related sarcopenia include: central and peripheral nervous system innervation, inflammatory effects, altered calorie and protein intake, and changes in the levels of circulating sex steroids [47,48,49]. There are substantial differences between the effects of androgens versus estrogens on skeletal muscle. The effects of either class of sex steroids depend on the level of muscle receptor expression and on the sensitivity of one muscle type versus another to these hormones [49].

There are conflicting epidemiological data regarding the gender specific prevalence rates of sarcopenic obesity in humans, ranging from 4.4% to 84% in men and 3.6% to 94% in women [50]. Adult men and women differ markedly in body composition; there are enormous differences in lipid storage capacities between the two genders and elevated amounts of endogenous fat having lipotoxic effects lead to the loss of muscle mass. Leptin might play a role in the differential regulation [51,52]. Women have more fat mass as well as lower absolute and relative muscle strength than men and are therefore more prone to develop obesity and poor strength, which contributes to sarcopenic obesity [53,54]. Men also have more appendicular muscle in the upper body than women. The grip strength declines more rapidly than the loss of upper limbs muscle mass in men but has the opposite tendency in women. Although the loss of muscle mass is present during the aging process of both men and women, this decrease does not occur at the same rate and age [55]. Gallagher et al. [56] found that gender greatly influences muscle mass and that, with age, men experience twice the decline in muscle mass as women do. Furthermore, some authors have suggested that during aging, there is also a decrease in the quality of the muscular tissue as a result of the selective loss of type 2 muscular fibers and to the increase in pro-inflammatory cytokines and neurogenic changes [6,7]

Fat infiltration into the mid-thigh muscle is a strong predictor of mobility limitations. The increase in fat infiltration correlates with overall body fatness and lower muscle strength [57]. There is low leg muscle mass and a greater fat infiltration of fat in muscle fat in men than in women [50].

### 3.1. Sarcopenia in Men

Testosterone (T) stimulates protein synthesis and inhibits protein degradation in the skeletal muscle cells [58]. It also increases replication and activation of the satellite cells in muscle fibers, which is reflected as a higher muscle mass [59]. Starting at the age of 35–40 years, circulating T concentration levels decrease by approximately 1–3% per year, where the most obvious clinical signs of relative deficiency in men are a decline in muscle mass, strength, and bone mass, and an increase in central body fat [60]. Progressive aging results in a decline of serum T concentrations and of adrenal androgens. These declines are a core physiological event in what is termed as andropause and can be clinically characterized by an increased fatigability and a decreased potency and libido [61]. Besides, the increase in muscle anabolism is associated with an increase in the expression of intramuscular mRNA for insulin-like growth factor 1 (IGF-1). This factor is a potent anabolic hormone that is associated with free T concentrations [61]. The fall of free T, low physical activity, cardiovascular disease, and the decrease of IGF-1 are significant predictors of muscle mass and fat mass in men and women [62]. T receptors are present in muscle and exert an anabolic effect. The decrease of these receptors in hypo-gonadal men constitutes a risk factor for the development of sarcopenia.

Estrogens could also participate in sarcopenia in men. Estrogens are produced through aromatization in men, a process that occurs in the limbic system and brain tissues, and by which the body converts T into estradiol (E2) [63]. Extra glandular aromatization of circulating androgens in young men is the origin of most of the E2 and nearly 20% of estrone. Direct secretion by the testicles provides for a smaller part of the circulating E2. In elderly men, most of the estrone is produced by the adrenals [64]. E2 deficiency in older men leads to hyper-gonadotropism, osteoporosis, and decreased muscle mass [65].

### 3.2. Sarcopenia in Women

The protective effect of estrogens on the heart, smooth, and skeletal muscle, as well as sustaining growth and maintaining muscular mass, is well documented [49]. Many details of the mechanisms of the action of estrogens still remain unknown; however, some evidence indicates they have anti-oxidant activity, they exert a membrane-stabilizing effect, and they may regulate the expression of downstream genes and molecular targets through their receptors [66].

The effects of estrogen upon muscle fibers are due to the stimulation of estrogen α-receptors. Through this pathway, estrogens depolarize the plasma membrane, eliciting electrical activity and intracellular calcium signals, which in turn enhance the muscle contractile function [67]. The α-receptors have been identified in human skeletal muscle and the force per unit cross sectional area is reduced during menopause and is accompanied by stretch force decreases that affect the activity of the myosin ATPase or the reuptake of calcium by the sarcoplasmic reticulum [68]. When estrogens are diminished, as in menopause, muscle mass and muscle strength are reduced.

Menopause occurs when all of the ovarian follicles have atrophied and plasma concentrations of estrogens fall; this generally happens between 35 and 58 years of age. During the first year of menopause, women lose an average of 80% of their estrogen and this is associated with an accelerated decline in muscle mass and strength [69,70]. The rate of decline accelerates after age 60 at a rate of approximately 3% annually, and at the same time, muscle strength declines annually by approximately 1–1.5% between 50 and 60 years and after. Women between 65–80 years old have twice the amount of non-contractile muscle tissue per unit cross-sectional area compared to younger women between 23 and 57 years [71]. The decline in estrogen levels in women also has catabolic effects on muscle, possibly as a result of its conversion to T [69].

Moreover, the loss of estrogen may promote the production of CRP, TNF-α, interleukin-1 (IL-1) and IL-6, causing inflammation and oxidative stress, and may have an indirect catabolic effect on muscle fibers [47,72]. In menopause, not only estrogen concentrations are affected but also other sex hormones involved in the regulation of different metabolic pathways, such as androgens, are also affected [73]. Even if women have a 20–25-fold lower circulating concentration of androgens when compared to men, these molecules are precursors for the estrogen synthesis and production, and determine, in part, the processes of maturation of the ovarian follicles [73]. However, androgens decline in older women and their loss is associated with impairments in sexual function, lean body muscle mass, muscle strength, performance, cognitive function, emotions, bone loss, and frailty [74]. In addition, during the fourth decade of life and before menopause, T levels in women diminish, reaching 50% of those observed in the third decade. When menopause is completed, T concentrations in women are nearly 15% of those found in premenopausal stages [75].

## 4. Repercussions of MetS-Related Sarcopenia in Other Diseases

The impact of sarcopenic obesity upon different diseases requires a global analysis to evaluate its association with the severity of the disease and with the coexistence of multiple morbidities; however, its presence increases health costs [76].

Although sarcopenia is usually associated with advanced age, its participation as a risk factor is not limited to this stage of life. Several diseases might predispose its appearance and it has recently been related to gene mutations. In elderly patients in which sarcopenia appears first, there is a functional limitation, probably conditioned by inflammation, which leads to chronic metabolic diseases such as insulin resistance, type 2 diabetes, and atherosclerosis [14,77]. However, in autoimmune systemic diseases with a chronic inflammatory state and risk of functional limitation, these diseases might induce sarcopenic obesity [78]. In chronic diseases, such as type 2 diabetes, sarcopenic obesity or presarcopenia precede the metabolic disease, while in type 1 diabetes, obesity sarcopenia appears during the course of the disease and is related to elevated levels of advanced glycation end products (AGEs) [79,80].

AGEs are a heterogeneous group of macromolecules that are formed by the nonenzymatic glycation of proteins, lipids, and nucleic acids. The damaging effects of AGEs are produced by: (1) alterations of the structure of intra- and extra-cellular proteins; and (2) by their union to their receptors (RAGEs), which form part of the immunoglobulin family and are found in plasma membranes, initiating second messenger cascades that modulate cellular function and induce oxidative stress and inflammatory processes [81]. AGEs may also play a role in sarcopenia through the upregulation of inflammation and endothelial dysfunction in the microcirculation of skeletal muscle. Indeed, the accumulation of AGEs in muscle fibers produces the cross-linking of muscle collagen and is associated with loss of muscle mass and strength [80,82,83].

The loss of body mass is associated with cardiovascular risk factors including vascular rigidity [84]. Loss of body mass increases arterial pressure and elevates the risk for hypertension or other diseases in addition to age and a progressive increase in fat mass and loss of function [85,86]. Sarcopenia and a low free fat mass index have been associated with poor exercise capacity, increased respiratory symptoms, and elevated mortality in patients with chronic obstructive pulmonary disease [87,88].

The presence of sarcopenia together with cardiac dysfunction is a risk factor that leads to 10% of patients developing cardiac failure. In patients with idiopathic dilated cardiomyopathy (IDCM), where there is also cardiac failure and a reduced left ventricular ejection fraction, there is a loss of body mass, even when they are young [89]. IDCM is a multifactorial consequence of genetic predisposition with the coexistence of other diseases such as hypertension, diabetes, chronic renal disease, viral infections, or alcohol ingestion.

There is often an interplay between the loss of function of the heart and kidneys in cardiorenal syndromes. The deterioration of one organ leads to the loss of function of the other, and in both cases, there is cachexia, a condition characterized by the loss of skeletal muscle tissue with or without fat loss, and which is related to obesity sarcopenia. The activation of the immune and neuroendocrine system and of oxidative stress contributes to the genesis of obesity sarcopenia, which may damage an organ. In patients with sustained cardiac inflammatory activity, all of these systems are upregulated leading to increased renal vascular resistance that alters renal perfusion. In turn, when there is renal damage, the pro-inflammatory cytokines may cause left ventricular systolic dysfunction, myocardiac cell death, endothelial dysfunction, and an increase in myocardic fibrosis, which finally affects the renal and cardiac functions.

When renal function is decreased with a low glomerular filtration rate and a high albumin/creatinin index, sarcopenia occurs [90]. Sarcopenia is present in at least one third of patients with chronic cirrhosis related to myosteatosis and is associated with an increase in mortality [91]. Obesity sarcopenia is present in hepatocellular carcinoma and constitutes an independent risk factor for mortality [92]. Although hepatic disease is accelerated in elderly people that ingest alcohol, a recent metaanalysis shows that there is no relation between alcohol consumption and sarcopenia [93,94]. In patients with renal chronic disease, there are mitochondrial and oxidative stress abnormalities, which are common in skeletal muscle. This finding might explain the muscular dysfunction and presence of sarcopenia in these patients [95]. 

## 5. Therapeutical Approaches

MetS-related sarcopenia is still poorly defined clinically and the mechanisms that might explain a common etiology with sarcopenia and obesity have not yet been well characterized. Therefore, there are no licensed treatments for it [14]. Only the preventive measures consisting of an equilibrated diet and the regular practice of exercise during all of the lifespan seem to be able to slow and reduce the decline in muscular mass and function present in sarcopenia [9,96]. Nevertheless, until now, the pharmacological procedures have not proven to be effective in its prevention [97].

### 5.1. Diet

An excess diet could exacerbate the age-related loss of muscle mass and further impair physical function. Diet-induced weight loss results in a decrease in both fat mass and fat-free mass, and so could help improve MetS-related sarcopenia. Despite a reduction in lean body mass, weight loss appears to be a suitable intervention for the treatment of sarcopenic obesity. 

There have been adjustments in the dietary guidelines to prevent sarcopenic obesity and to help the medical professional in the management of weight loss in the presence of sarcopenic obesity, which are founded on intensive research regarding sarcopenia and sarcopenic obesity. Sufficient protein intake (25–30 g of protein per meal) is important for optimizing the muscle protein synthetic response [98,99]. A diet relatively low in carbohydrates may also be advisable as the co-ingestion of carbohydrates has been shown to exert negative effects on muscle protein turnover in the elderly [100,101].

In inflammatory chronic cardiac diseases, a diet optimization might help the energetic balance, giving as a result an increase in the non-fat tissue mass. Diet changes improve respiratory mechanics and oxygen acquirement and also benefit the function of the immune system [102].

Indeed, supplementation with leucine, which is the most potent branched-chain amino acid for increasing protein synthesis, might be helpful for preventing sarcopenia [103]. An association was found between leucine supplementation and increased muscle protein synthesis independently of ingestion of other amino acids in older adults [104]. Leucine is a potent activator of the mammalian target of the rapamycin (mTOR) nutrient and energy-sensing signaling pathway. Furthermore, the leucine supplementation has been associated with a decrease in serum TNF-α levels and improved insulin sensitivity [98,105,106].

The consumption of natural antioxidant supplements (flavonoids and polyphenols) is rising in adults to treat obesity and MetS. They have been found to have a role in the reduction of cardiovascular diseases, cancer, and neurodegenerative disorders. Beneficial effects are attributed to their potent antioxidant and anti-inflammatory action, and the activation of a histone NAD^+^-dependent deacetylase sirtuin 1 (SIRT 1) [107,108]. SIRT1 regulates the expression of some antioxidant enzymes and also deacetylates and activates PGC-1α, which inhibits muscular atrophy. In this regard, resveratrol and quercetin treatment may be useful for protecting against obesity-induced sarcopenia [109,110].

### 5.2. Exercise

Multiple combined exercise and mild caloric restriction markedly attenuate the symptoms of sarcopenic obesity [1]. Different protocols using several exercise programs increase muscular mass (muscular hypertrophia), force development, and functional capacity. High intensity exercise is generally considered to be the preferred protocol to counteract the age-associated muscular decline when compared to low- and moderate-intensity exercise [110,111,112].

PGC-1α is a key mediator of the beneficial effects of endurance exercise and constitutes a molecular target that might be a promising candidate for the alleviation of both metabolic inefficiency and sarcopenia. Protection against mitochondrial disorders (apoptosis, oxidative damage, etc.) might be caused by an increase in the production of PGC-1α [113]. Increased expression of PGC-1α in muscle improves metabolic fitness and prevents sarcopenia in aging mice [114]. 

There are several intracellular signaling pathways that may contribute to eliciting the exercise-induced PGC-1α gene expression response including calcium signaling, AMPK and MAPK signaling, ROS-mediated regulation, and β-adrenergic signaling [115]. AMPK activation could have a net beneficial effect in insulin resistance and sarcopenia. There is an inverse relationship between activated AMPK phosphorylation and load-induced muscular hypertrophy in rodents [116]. AMPK stimulates myofibrillar protein degradation by increasing the expression of forkhead transcription factors of the O class (FOXO), which play important roles in metabolism, cellular proliferation, stress resistance, and apoptosis [117]. It causes down-regulation of the mTOR pathway, diminishing protein synthesis [118]. Differentiation of satellite cells (stem cells present in adult skeletal muscle) is decreased by liver kinase b1, one of AMPK’s upstream kinases [119]. Moreover, activation of PGC-1α results in increased secretion of a novel myokine called irisin. The role of this polypeptidic hormone is a matter of debate. Some authors have proposed that it is the mediator of the beneficial effects of exercise by inducing mitochondrial biogenesis and oxidative metabolism and by improving insulin sensitivity. However, the role of irisin in animal models and humans is still questionable [120,121,122].

Metformin is a drug that has been used for the treatment of type-2 diabetes with anti-inflammatory properties since it mimics some of the effects of exercise by stimulating the same metabolic pathways (AMPK). However, whether metformin is an effective treatment for sarcopenia is still a matter of debate [123].

### 5.3. Insulin and Insulin-Like Growth Factor-1

Insulin and insulin-like growth factor-1 (IGF-1) have predominant metabolic and anabolic effects on muscle, constituting powerful anabolic signals [124]. The activation of PI3K pathways has positive effects on muscle size and metabolism. Insulin significantly stimulates muscle protein synthesis in young but not older subjects.

In sarcopenia, there is a reduced muscle protein synthesis in response to nutrients or insulin and a reduced insulin mediated suppression of proteolysis, which has been referred to as “anabolic resistance.” Elderly individuals of normal muscle mass also show resistance to the anabolic action of insulin, which may precede the physical expressions of sarcopenia [125,126,127]. Differential insulin resistance with respect to glucose, protein, and lipid metabolism can develop with aging and sarcopenic obesity. Many elderly persons respond to insulin by modifying glucose metabolism, but protein synthesis is not affected by the hormone [128]. When elevated adiposity is present, the effectiveness for inducing muscle protein synthesis increases of insulin, essential amino acids, and resistance exercise is decreased [129,130,131]. The insulin-mediated increase of muscle mass is mediated by the activation of p38 MAPK and mTOR/p70S6 kinase, and stimulation of mRNA translation [126,132].

Treatment with the insulin-sensitizing thiazolidinedione drug rosiglitazone leads to an improvement in muscle mass. Although impairment of the Akt-mTOR pathway in muscle does not seem to occur during aging in humans or mice, stimulation by rosiglitazone of PPAR-γ could also result in the activation of the Akt-mTOR cellular signaling pathways having a beneficial effect upon insulin resistance and muscle mass [133].

### 5.4. Growth Hormone

Growth of multiple target tissues, including skeletal muscle, is regulated by growth hormone (GH), which is a single-chain peptide produced and secreted by the somatotrophs of the anterior pituitary gland [134]. After 30 years of age and in aged men, circulating GH levels decline progressively; GH secretion per day is 5- to 20-fold lower in elderly people than the secretion found in young adults [135,136]. Hormonal supplementation for the elderly has been tested in a large population; however, it was not effective to treat sarcopenia and it showed minor side effects [136,137,138]. Its use is not favored by the fact that high circulating levels of free fatty acids, which are present in obesity, inhibit GH production and decrease plasma levels of IGF-1 [139,140]. A recent study showed that sarcopenic obese subjects had depressed GH secretion when compared to obese persons [141]. Makimura et al. [142] recently reported the effects of a GH-receptor analog that reduced fat mass and increased lean body mass in obese individuals was not associated with abnormalities in glucose homeostasis or other adverse events compared to placebo [143]. 

### 5.5. Sex Hormones

#### 5.5.1. Androgens

Testosterone (T) increases muscle protein synthesis, and its effects on muscle are modulated by several factors including genetic background, nutrition, and exercise [144,145].

Obese individuals tend to have lower T levels [146]. In males, levels of T decrease by 1% per year and those of bioavailable T by 2% per year from age 30 [147]. In women, T levels drop rapidly from 20 to 45 years of age [148]. High levels of these anabolic hormones are positively associated with elevated muscle strength and may therefore contribute to muscle improvement in obese individuals [149,150,151]. In young men, low T secretion results in decreased muscle mass and strength, and T replacement therapy increases muscle mass, increases the sensibility of T receptors and restores muscle strength [152,153]. Sinha-Hikim et al. [154] demonstrated that supra-physiological doses of T can induce increase in muscle size and strength in younger men without concomitant exercise. 

Hormone therapy with T can help preserve muscle strength but it carries a certain risk, especially when the treated population is unhealthy or when supra-physiologic doses are administered rather than replacement doses [155]. Calculating the correct concentration of hormone per individual considering the appropriate threshold without collateral damage, can be difficult since T may have effects on different organs. For example, T replacement has been associated with higher rates of prostate cancer, prostate specific antigen, erythrocytosis cardiovascular events, acne, oily skin, reduced sperm production, and fertility [156]. Orally administered androgens are known to be hepatotoxic and may promote hepatic steatosis which is associated with dyslipidemia and increased very low density lipoprotein- triglyceride (VLDL-TG) secretion [157]. However, lower T concentrations in older men are associated with higher atherosclerosis and myocardial infarction [158]. The above suggests that the threshold in the concentration of T is very important to obtain the beneficial effects on metabolic pathways related with this hormone [159].

Nevertheless, androgen therapy may also be linked with improved insulin sensitivity [160]. A selective androgen receptor could be developed in the future that might not have many of these side effects. Candidate drugs having beneficial effects on insulin sensitivity, muscle mass, and strength have been found in preclinical and phase II trials [161,162].

#### 5.5.2. Estrogens

Numerous studies have revealed that estrogens mediate the attenuation of infiltration of inflammatory cells, such as neutrophils and macrophages, into skeletal muscles of rats following exercise or injury. It also plays a significant role in stimulating muscle reparation and generative processes including the activation and proliferation of satellite cells. However, the mechanisms by which E2 exerts influence on damaged muscle and can influence the force generating capacity of skeletal muscle are still unknown [163]. An E2 reduction is associated with decreased muscle size and a decline in force-generating capacity; this can be prevented using hormone replacement treatment [68]. Lowe et al. showed that estrogen has beneficial effects on muscle strength in postmenopausal women. E2 replacement therapy also has beneficial effects against menopause-related obesity sarcopenia [164].

However, E2 effects on muscle structure and contractile function in humans are controversial and depend on age, muscle size, and muscular fiber type [165]. For example, T or E2 are potent skeletal muscle protein anabolic agents in men and women. Administration for 3 weeks in obese but otherwise healthy premenopausal women did not affect plasma lipid kinetics and concentrations [157].

The beneficial effects of estrogen therapy were associated to an increase of pro-anabolic markers, such as MyoD, myogenin, Myf5, and the greater suppression of proteolytic markers, such as FOXO3A, as well as the negative growth regulator, myostatin. These beneficial effects are even more evident when combined with exercise [166].

### 5.6. Myostatin Inactivation

Myostatin (MSTN) was first discovered during screening for novel members of the transforming growth factor-β (TGF-β superfamily). MSTN is a potent negative regulator of muscle growth [167]. Beneficial effects on metabolism, adiposity, and insulin sensitivity have been found with low levels of MSTN. There was elevated muscle glucose utilization and insulin sensitivity linked to increased lean mass and diminished fat mass in MSTN-null mice and in mice treated with a soluble receptor to MSTN, namely activin receptor type IIB (ActRIIB). MSTN leads to receptor mediated phosphorylation of Smads 2 and 3, and binds to Smad 4. Increased Smad2/3/4 signaling inhibits the Akt/mTORC1 pathway, thus leading to protein degradation and muscle atrophy (Figure 1) [168,169,170].

Mutations in MSTN cause significant hypertrophy and/or hyperplasia in developing animals [167]. The inhibition of MSTN induced by gene manipulation or neutralizing antibodies improves sarcopenic obesity by increasing skeletal muscle mass and improving glucose homeostasis [1]. Moreover, when MSTN is inactivated, activation of AMPK, which leads to increased lypolisis, elevated fatty acid oxidation in peripheral tissues, and a high expression of brown adipocyte markers in white adipose tissue [171,172].

Overexpression of the MSTN propeptide and sequestration of the active peptide enhances skeletal muscle glucose disposal due to increased muscle mass, implying that additive or synergistic mechanisms are in operation [14]. Although the inhibition of MSTN activity as a therapeutic approach has not been effective, the use of antisense-mediated destructive exon skipping is being evaluated. It seems to preserve muscle mass in a mouse model of Duchenne muscular dystrophy [173].

MSTN inhibition induce a reduction of fat in obesity and osteoporosis and has also been suggested for other diseases in which cachexia is present such as cancer, acquired immune deficiency syndrome (AIDS), obstructive chronic pulmonary disease, and renal failure [174]. Moreover, there was no amelioration in muscle strength or function in muscular dystrophy patients in one phase I/II trial of a MSTN antibody [175]. There were also no important improvements in muscle strength by MSTN inhibition in muscular dystrophy patients in another study, although there was an amelioration in muscle function at a cellular level [176].

### 5.7. Urocortins

The central nervous system and peripheral tissues express neuropeptide ligands for the corticotrophin-releasing factor receptor 2 (CRF2R) known as urocortins (Ucns). This family of proteins plays different roles in metabolic functions, adaptive stress being one of them. Modulation of CRFR2 or its ligands may improve muscle mass and metabolism by activating the hypothalamic–pituitary–adrenal (HPA) axis [177]. Skeletal muscle has high levels of Ucn2 and CRFR2 [178]. Knockout mice for Ucn2 or Crfr2 are resistant to diet-induced obesity and Ucn2 knockouts have increased muscle mass [179,180]. Overexpression of Ucn3 also results in mice with muscular hypertrophy [180]. Short-term overexpression of Ucn3 in rat muscle increased glucose disposal, elevated levels of glucose transporter expression, and phosphorylation of both AMPK and insulin signaling molecules. Muscle mass increased afterwards in these mice [181].

Since Ucns or CRF2R agonists have potentially beneficial effect in preserving skeletal muscle mass/function in a cachexia state linked to other diseases, such as cancer, it would be desirable to test their use for the treatment of sarcopenic obesity.

### 5.8. Vitamin D

One of the new roles described for vitamin D is the maintenance of muscle mass and insulin sensitivity [182]. Although dietary insufficiency of this vitamin impairs muscle strength as a result of hypophosphatemia in rats; in humans, contradictory results have been obtained [183]. Insulin sensitivity either improves or is unaffected by vitamin D supplementation [184,185]. Vitamin D supplementation was reported to increase muscle fiber size in immobile older women; however, supplementation in individuals with vitamin D deficiency improved muscle strength, but not muscle mass [186,187]. In knockout mice for the vitamin D receptor (Vdr), there was reduced muscle size, impaired motor activity, and abnormal muscle development [188,189]. In addition, Vdr-null mice are leaner, but insulin resistant [190]. The relationship between vitamin D status and frailty is largely mediated by the development of sarcopenia. A minimum serum 25-hydroxyvitamin D level of 75 nmol/L is proposed for frail elderly patients and the doses necessary to reach this target are between 800 and 2000 IU/day [191]. Thus, the protective role of vitamin D for sarcopenic obesity remains unclear and studies in order to establish a role for vitamin D on the treatment of sarcopenia are still needed [192].

### 5.9. Angiotensin 1–7 and Angiotensin-Converting Enzyme Inhibitors

The renin–angiotensin system (RAS) is an important regulator of skeletal muscle mass. The classical axis (angiotensin (AT) II, angiotensin converting enzyme (ACE), AT1 receptor, and AT2 receptor) and the non-classical RAS axis (angiotensin 1–7, ACE2, and Mas receptor), have been found in skeletal muscle and could play a role in the regulation of muscle function by a differential expression of the biochemical and/or metabolic features of the fibers [193].

Some authors have addressed the metabolic actions of RAS in skeletal muscle. Activation of the classical RAS causes deleterious effects in skeletal muscle, including muscle wasting. In contrast, angiotensin 1–7 produces beneficial effects in skeletal muscle by downregulating the catabolic pathway of sarcomere proteins and preventing the atrophic effects induced by TGF-β [194]. Angiotensin 1–7 treatment was able to maintain muscle strength and prevented decreases in muscle diameter and mass by activating IGF-1 and Akt pathways that also might improve insulin resistance in skeletal muscle [195]. Moreover, angiotensin 1–7 had an ameliorating effect on insulin resistance, hypertriglyceridemia, fatty liver, inflammation, obesity, and oxidative stress in MetS models [196]. Thus, some authors have suggested the use of angiotensin-converting enzyme inhibitors to modulate RAS, favoring the production of angiotensin 1–7, thus contributing to the change in body composition and preventing the development of sarcopenia and simultaneously ameliorating the MetS pathophysiologic parameters [108,196,197,198]. Mechanisms of action of some therapeutic agents for MetS-related sarcopenia are summarized in Table 1.

## 6. Conclusions

The rising prevalence of MetS coupled with the age-related decline in muscle mass results in the high prevalence of sarcopenia. A vicious cycle between the loss of muscle and the accumulation of intramuscular fat might be associated with MetS via a complex interplay of factors including nutritional intake, physical activity, body fat, oxidative stress, proinflammatory cytokines, insulin resistance, hormonal changes, and mitochondrial dysfunction. MetS-related sarcopenia has important health repercussions. Although currently, there is no consensus on the treatment of sarcopenia, lifestyle changes including diet and power exercise seem to be the best option.

## Figures and Tables

**Table 1:** The beneficial physiological effects of some treatments for metabolic syndrome-related sarcopenia. (↓) inhibition, down-regulation, or reduction of activity; (↑) stimulation, induction, or increase of activity.

Therapeutical Agent	Effects/Molecular Target	References
Protein intake	↑protein turnover ↑oxygen acquirement	Volpi et al. [100] Anad et al. [102]
Leucine	↑energy-sensing signaling ↑insulin sensitivity	Paddon-Jones et al. [98] Drummond et al. [105] Solerte et al. [106]
Flavonoids and polyphenols	↓muscular atrophy ↑Sirt1	Le et al. [109] Hori et al. [110]
Resistance exercise	↑Metabolic fitness: ↑PGC-1α ↓insulin resistance: ↑AMPk ↓muscular hypertrophy	Law, et al. [114] Thompson, et al. [116]
Insulin	↑muscle mass and metabolism ↑MAPk ↑mTOR/p70S6k	Guillet et al. [126] Fuijita et al. [128]
Rosiglitazone	↑muscle mass ↑Akt ↑mTOR	Sandri et al. [133]
Sex hormones	↑muscle size and force ↑insulin sensitivity	Sinha-Hikim et al. [154] Traish et al. [160] Dalton et al. [161] Tiidus et al. [166]
Myostatin inactivators	↑lean mass ↓fat mass ↑glucose homeostasis	Sakuma et al. [1] Zhang et al. [171] Zhang et al. [172]
Urocortins	↑muscle mass and metabolism ↑HPA axis ↑insulin signaling pathway	Hinkle et al. [173] Roustit et al. [177]
Vitamin D	↑muscle mass/force ↑insulin sensitivity	Bates et al. [182] Ceglia et al. [186] Narvaez et al. [190]
Angiotensin 1–7	↓catabolic pathway ↓insulin resistance: ↑Akt ↑IGF-1 ↓hypertriglyceridemia	Morales et al. [195] Marcus et al. [196] Carter et al. [197] Sartiani et al. [198]
